# Clinical performance of the BioFire Blood Culture Identification 2 panel for microorganism species identification and resistance gene detection in blood culture-positive specimens

**DOI:** 10.1371/journal.pone.0350768

**Published:** 2026-06-05

**Authors:** Haruki Naruse, Noriyuki Watanabe, Sachie Koyama, Sachi Tanaka, Yoshitada Taji, Yasuhiro Ebihara

**Affiliations:** 1 Clinical Laboratory, Saitama Medical University International Medical Center, Saitama, Japan; 2 Department of Laboratory Medicine, Saitama Medical University International Medical Center, Saitama, Japan; Rutgers Biomedical and Health Sciences, UNITED STATES OF AMERICA

## Abstract

**Introduction:**

Bloodstream infections are life-threatening, and the rapid identification of pathogens and resistance genes is essential for the administration of appropriate antimicrobial agents. The BioFire Blood Culture Identification 2 (BCID2) panel on the FilmArray multi-parameter genetic analyzer is a fully automated PCR test that rapidly identifies species and resistance genes. Here, we compared the performance of the Filmarray BCID2 panel (BCID2 method) with the conventional method.

**Methods:**

Among the blood culture-positive specimens submitted between January 2023 and November 2024, this study analyzed 201 specimens that contained the target microorganisms of the BCID2 panel. In our laboratory, after subculturing the culture medium obtained from a positive blood-culture bottle, we perform species identification using mass spectrometry and drug susceptibility testing (the conventional method). We compared the results of the BCID2 method with those of the conventional method.

**Results:**

Concordance between the BCID2 and conventional methods was found in 152 of the 161 monomicrobial specimens (94.4%) and in 31 of the 40 polymicrobial specimens (77.5%). The 18 specimens that were discordant were mostly matched at the genus level, but the BCID2 method also detected other microorganisms that were not identified by the conventional method. Resistance genes were identified in 57 of the 61 matched specimens (93.4%).

**Conclusion:**

The BCID2 method exhibits excellent identification results and resistance gene detection rates, suggesting that it is a reliable and rapid diagnostic test system for bloodstream infections.

## Introduction

Bloodstream infections are severe conditions with high morbidity and mortality rates [1,2]. Rapid species identification is crucial for the appropriate administration of appropriate antimicrobial agents. It is also important to know whether the identified pathogens harbor drug-resistance genes because the prevalence of drug-resistant strains has been rising [3].

The gold standard for the diagnosis of bloodstream infections is blood culture. Generally, when a blood-culture bottle is flagged as positive, an aliquot is cultured onto solid media and pathogens are identified based on the colonies that grow. This is the conventional method used in our laboratory. These colonies are also subjected to antimicrobial susceptibility testing. If drug resistance is suspected, the disk-diffusion method and/or genetic analysis is used to detect drug-resistance genes.

Rapid diagnostic test systems that can be directly performed using positive blood-culture specimens include the BioFire FilmArray Torch System (bioMérieux, Marcy l’Étoile, France), a fully automated genetic analyzer [4–6]. This system identifies microorganisms directly from clinical specimens through nucleic acid extraction, multiplex polymerase chain reaction (PCR), and post-PCR DNA melting curve analysis [7,8].

The BioFire Blood Culture Identification (BCID) panel (bioMérieux) was originally designed to identify 24 microorganisms and 3 antimicrobial resistance genes [4–6], while the recently released second-generation BCID2 panel identifies 33 microorganisms and 10 antimicrobial resistance genes [9–16].

In the present study, we analyzed the performance of the FilmArray BCID2 panel (the BCID2 method) by comparing its species identification results with those of the conventional method. In addition, we assessed the detection of drug-resistance genes between these methods in isolates containing drug-resistance genes.

## Materials and methods

### Study design

This retrospective single-center analysis was performed at Saitama Medical University International Medical Center. The study was approved by the Institutional Review Board of Saitama Medical University International Medical Center (#2022−023) and conformed to the tenets of the Declaration of Helsinki. The need for informed consent was waived because of the retrospective nature of the study and the anonymity of the data. The inclusion criteria for this study were specimens collected between January 2023 and November 2024 that had positive blood cultures and in which microorganisms included in the BCID2 panel were detected using conventional methods. After receiving Institutional Review Board approval, the data were extracted from the hospital’s electronic medical records between January 2023 and November 2024. During the data-collection period, we had access to information that could identify patients. However, all data was anonymized prior to analysis, and thus no identifying information was used in the analysis.

### Clinical specimens

A total of 212 positive blood-culture specimens satisfied the inclusion criteria and were included in the analysis. These specimens were collected from patients admitted to Saitama Medical University International Medical Center between January 2023 and November 2024. Of the 212 specimens, the microorganisms identified by the conventional method in 11 specimens were not included in the BCID2 panel, and these specimens were excluded from the study. Thus, 201 positive blood-culture specimens were analyzed.

### Species identification analysis

In the conventional method, culture media from blood-culture bottles (BACTEC^®^ Plus Aerobic/F, Anaerobic/F; BD Biosciences, Franklin Lakes, NJ) flagged as positive in the BACTEC FX system (BD Biosciences) were subcultured on 5% sheep blood agar (Kyokuto Pharmaceutical Industrial, Tokyo, Japan) and MacConkey agar medium (Eiken Chemical, Tokyo, Japan). The colonies formed on the plate were used for species identification by MALDI-TOF MS on a Microflex LT platform (MALDI Biotyper Ver. 8.0; Bruker, Billerica, MA), according to the manufacturer’s instructions. Mass spectra were analyzed using Bruker Biotyper ver. 3.4 software and library (7854 isolates; Bruker). Scores ≥2.0 were considered acceptable.

The samples used for the BCID2 method were the residual clinical specimens after the conventional method was performed; they were not collected specifically for the BCID2 method. The BCID2 method was performed within 24 h using the FilmArray Torch System, according to the manufacturer’s instructions. Briefly, 200 μL of culture media was collected from a positive blood-culture bottle and lysed with 500 μL specimen-dilution buffer in the provided specimen vial. This specimen vial was injected into a BCID2 pouch pre-rehydrated with hydration solution. The pouch was inserted into the FilmArray system to start the identification procedure and detect drug-resistance genes.

### Antimicrobial susceptibility testing

Individual colonies from the agar subculture were picked, and antimicrobial susceptibility testing was conducted using a BD Phoenix M50 platform (BD Biosciences). If the identified pathogen possibly contained drug-resistance genes according to antimicrobial susceptibility testing, the disk-diffusion method was performed. The AmpC/ESBL Differentiation Disk (Kanto Chemical, Tokyo, Japan) was used according to the manufacturer’s instructions to identify extended-spectrum β-lactamase (ESBL)-producing bacteria, and the genotype of ESBL was determined using a Cica Geneus^®^ ESBL Genotype Detection KIT2 (Kanto Chemical). The presence or absence of the ESBL gene was confirmed using Sanger sequencing with CTX-M primer and Mighty Amp™ DNA Polymerase Ver. 3 (Takara, Kusatsu, Shiga). Carbapenem-resistance genes were examined using a Cica Geneus^®^ Carbapenemase Genotype Detection KIT2 (Kanto Chemical), according to the manufacturer’s instructions. *Staphylococcus aureus* was determined to be methicillin-resistant *S. aureus* (MRSA) when the MIC value of oxacillin was ≥ 4 μg/mL or that of cefoxitin was ≥ 8 μg/mL. *Staphylococcus* spp. other than *S. aureus* or *S. lugdunensis* were determined to be methicillin-resistant coagulase-negative staphylococci (MRCNS) when the MIC value of oxacillin was ≥ 0.5 μg/mL (CLSI M100-S29). The *mec*A genotypes of MRSA were identified using a Cica Geneus^®^ Staph POT KIT (Kanto Chemical), according to the manufacturer’s instructions.

### Definition of consistency

In the bacterial species identification, considering the detection limit of the BCID2 method, the results were considered to be consistent when the results of the BCID2 and conventional methods matched at the genus or order (Enterobacterales) level. The results were considered inconsistent when the BCID2 method identified species only at the genus level, given that this method can identify pathogens at the species level. When a resistant bacterium was identified using the conventional method, the results were considered consistent if the same bacterium was identified using the BCID2 method and resistance genes were also detected in the resistant bacterium.

### Data analysis

The results of the BCID2 method were considered to be a true positive (TP) or true negative (TN) when it agreed with the results of the conventional method. The results were considered to be a false positive (FP) or false negative (FN) when it disagreed with the results of the conventional method. Positive percent agreement (PPA) was defined as the proportion of culture-positive cases that were also positive using the BCID2 method, while negative percent agreement (NPA) was defined as the proportion of culture-negative cases that were also negative using the BCID2 method.

## Results

### Species identification

The overall concordance rate between the conventional and BCID2 methods was 91.0% (183 of 201 specimens). Among the 201 specimens, 161 monomicrobial and 40 polymicrobial specimens were identified using the conventional method.

The concordance rate between the conventional and BCID2 methods was 94.4% (152 of 161). The discordance was due to the fact that the BCID2 method identified a different bacterial species (FP) in addition to those identified using conventional method (Tables 1 and 2).

**Table 1:** Concordance in monomicrobial identification between the conventional and BCID2 (n = 170).

	No. of samples with BCID2/conventional methods					
	TP	FP	FN	TN		
Species	+/+	+/−	−/+	−/−	PPA	NPA
Gram-positive cocci						
*Staphylococcus* spp.	54	5	0	111	100%	96%
*Staphylococcus aureus*	35	0	0	135	100%	100%
*Staphylococcus epidermidis*	13	4	0	153	100%	97%
*Staphylococcus lugdunensis*	0	1	0	169	−	99%
Other *Staphylococus* spp.	6	0	0	164	100%	100%
*Streptococcus* spp.	20	0	0	150	100%	100%
*Streptococcus agalactiae*	6	0	0	164	100%	100%
*Streptococcus pneumoniae*	3	0	0	167	100%	100%
*Streptococcus pyogenes*	1	0	0	169	100%	100%
Other *Streptococcus* spp.	10	0	0	160	100%	100%
*Enterococcus faecalis*	5	0	0	165	100%	100%
*Enterococcus faecium*	7	0	0	163	100%	100%
						
Gram-positive rods						
*Listeria monocytogenes*	1	0	0	169‌‌	100%	100%
						
Gram-negative rods						
*Acinetobacter calcoaceticus-baumanii* complex	1	0	0	169	100%	100%
Enterobacterales	45	1	0	124	100%	99%
*Enterobacter cloacae* complex	5	0	0	165	100%	100%
*Escherichia coli*	22	0	0	148	100%	100%
*Klebsiella aerogenes*	2	0	0	168	100%	100%
*Klebsiella oxytoca*	1	1	0	168	100%	99%
*Klebsiella pneumoniae*	9	0	0	161	100%	100%
*Proteus* spp.	0	0	0	170	−	100%
*Salmonella* spp.	1	0	0	169	100%	100%
*Serratia marcescens*	1	0	0	169	100%	100%
Other Enterobacterales	4	0	0	166	100%	100%
*Haemophilus influenzae*	0	0	0	170		100%
*Pseudomonas aeruginosa*	8	0	0	162	100%	100%
*Stenotrophomonas maltophilia*	1	0	0	169	100%	100%
*Bacteroides fragilis*	5	0	0	165	100%	100%
*Neisseria meningitidis*	0	0	0	170		100%
						
Yeast-like fungi	15	3	0	152	100%	98%
*Candida albicans*	8	0	0	162	100%	100%
*Candida auris*	0	0	0	170		100%
*Candida glabrata*	2	0	0	168	100%	100%
*Candida krusei*	0	0	0	170	−	100%
*Candida parapsilosis*	5	0	0	165	100%	100%
*Candida tropicalis*	0	3	0	167	−	98%
*Cryptococcus neoformans*	0	0	0	170	−	100%

TP, true positive; FP, false positive; FN, false negative; TN, true negative PPA, positive percent agreement; NPA, negative percent agreement. PPA and NPA were calculated by comparing the results of the conventional culture (reference method) with those of the BCID2. The conventional and BCID2 methods identified 161 pathogens and 170 pathogens from 161 specimens, respectively. The BCID2 method identified the same bacteria as the conventional method (+/+) as well as other bacterial species (+/−). See the text for details of the data analysis.

**Table 2:** Specimens identified as monomicrobial using the conventional method but identified differently using the BCID2 method (n = 9).

No.	ID using the conventional method	ID using the BCID2 method	
		Concordant microorganism	Discordant microorganism
1	*Staphylococcus aureus* (MRSA)	*Staphylococcus aureus* (*mec*A/C and MREJ)	*Staphylococcus epidermidis* (*mec*A/C)
2	*Staphylococcus caprae (MRCNS)	*Staphylococcus* spp. (*mec*A/C)	*Staphylococcus epidermidis*
3	*Enterococcus faecalis*	*Enterococcus faecalis*	*Staphylococcus epidermidis* (*mec*A/C)
4	*Citrobacter koseri	Enterobacterales	*Staphylococcus epidermidis*
5	*Enterococcus faecalis*	*Enterococcus faecalis*	*Staphylococcus lugdunensis*
6	*Candida parapsilosis*	*Candida parapsilosis*	*Candida tropicalis*
7	*Candida parapsilosis*	*Candida parapsilosis*	*Candida tropicalis*
8	*Escherichia coli*	*Escherichia coli*	*Candida tropicalis*
9	*Klebsiella pneumoniae*	*Klebsiella pneumoniae*	*Klebsiella oxytoca*

ID, species identification; MRSA, methicillin-resistant *S. aureus*; MRCNS, methicillin-resistant coagulase-negative Staphylococci.

* This microorganism is not a target of the panel. *Staphylococcus caprae* and *Citrobacter koseri* belong to *Staphylococcus* spp. and Enterobacterales, respectively. These combinations were judged to be concordant, based on the definition of concordance and discordance (see main text).

In this study, we identified 170 microorganisms from 161 specimens. Specifically, the concordance rates were 94.5% (86 of 91 isolates) for Gram-positive cocci, 100% (1 of 1) for Gram-positive rods, 98.3% (59 of 60) for Gram-negative rods, and 83.3% (15 of 18) for yeast-like fungi (Table 1). Based on these results, the PPA and NPA were calculated. PPA was 100% and NPA ranged from 96% to 100%, demonstrating that the BCID2 method exhibited excellent performance.

Nine specimens were identified as containing a single microorganism when using the conventional method but as polymicrobial when using the BCID2 method. In all 9 discordant specimens, the BCID2 method identified other pathogens in addition to the same pathogens identified by the conventional method (Table 2). The additional pathogens that were detected only by the BCID2 method were 5 CNS (4 *S. epidermidis* and 1 *S. lugdunensis*), 1 enterobacterium (*Klebsiella oxytoca*), and 3 yeast-like fungi (all *Candida tropicalis*). In 1 specimen (Table 2 #4) in which *Citrobacter koseri* was identified by the conventional method and Enterobacterales was identified by the BCID2 method, the identification result was determined to be concordant between *C. koseri* and Enterobacterales based on the above-mentioned definition of consistency.

The conventional method identified 15 fungi in 15 specimens. The BCID2 method also identified these 15 fungi as well as 3 others (Table 1). Of the 2 discordant specimens, a single fungus was identified as *C. parapsilosis* by the conventional method while 2 fungi (*C. parapsilosis* and *C. tropicalis*) were identified by the BCID2 method (Table 2 #6 and 7). In 1 specimen, the conventional method identified *Escherichia coli* alone, while the BCID2 method additionally identified *C. tropicalis* (Table 2 #8).

Polymicrobial pathogens were identified in 40 specimens by the conventional method, and 31 specimens (77.5%) were concordant between the conventional and BCID2 methods (Table 3). Of the 9 discordant specimens (Table 4), the BCID2 method identified 8 other microorganisms, in addition to the microorganism identified by the conventional method. The 2 microorganisms identified by the conventional method were the BCID2 off-panel targets (Table 4 #2 [*C. freundii*] and #3 [*Aeromonas caviae*]).

**Table 3:** Concordance in polymicrobial identification between the conventional and BCID2 methods (n = 98).

	No. of samples with BCID2/conventional methods					
	TP	FP	FN	TN		
Species	+/+	+/−	−/+	−/−	PPA	NPA
Gram-positive cocci						
*Staphylococcus* spp.	25	2	0	71	100%	97%
*Staphylococcus aureus*	7	0	0	91	100%	100%
*Staphylococcus epidermidis*	8	2	0	88	100%	98%
*Staphylococcus lugdunensis*	3	0	0	95	100%	100%
Other *Staphylococus* spp.	7	0	0	91	100%	100%
						
*Streptococcus* spp.	3	2	1	92	75%	98%
*Streptococcus agalactiae*	3	0	1	94	75%	100%
*Streptococcus pneumoniae*	0	0	0	98	−	100%
*Streptococcus pyogenes*	0	0	0	98	−	100%
Other *Streptococcus* spp.	0	2	0	96	−	98%
*Enterococcus faecalis*	15	0	1	82‌‌	94%	100%
*Enterococcus faecium*	5	0	0	93‌‌	100%	100%
						
Gram-positive rods						
*Listeria monocytogenes*	0	0	0	98	−	100%
						
Gram-negative rods						
*A. calcoaceticus-baumanii* complex	2	0	0	96	100%	100%
Enterobacterales	24	3	1	70	96%	96%
*Enterobacter cloacae* complex	4	0	0	94	100%	100%
*Escherichia coli*	8	1	0	89	100%	99%
*Klebsiella aerogenes*	0	0	0	98	−	100%
*Klebsiella oxytoca*	3	1	0	94	100%	99%
*Klebsiella pneumoniae*	4	1	0	93	100%	99%
*Proteus* spp.	0	0	0	98	−	100%
*Salmonella* spp.	0	0	0	98	−	100%
*Serratia marcescens*	1	0	1	96	50%	100%
Other Enterobacterales	4	0	0	94	100%	100%
*Haemophilus influenzae*	0	0	0	98	−	100%
*Pseudomonas aeruginosa*	7	2	0	89	100%	98%
*Stenotrophomonas maltophilia*	0	1	0	97	−	99%
*Bacteroides fragilis*	2	2	0	94	100%	98%
*Neisseria meningitidis*	0	0	0	98	−	100%
						
Yeast-like fungi						
*Candida albicans*	0	0	0	98	−	100%
*Candida auris*	0	0	0	98	−	100%
*Candida glabrata*	0	0	0	98	−	100%
*Candida krusei*	0	0	0	98	−	100%
*Candida parapsilosis*	0	0	0	98	−	100%
*Candida tropicalis*	0	0	0	98	−	100%
*Cryptococcus neoformans*	0	0	0	98	−‌‌	100%

TP, true positive; FP, false positive; FN, false negative; TN, true negative; PPA, positive percent agreement; NPA, negative percent agreement. PPA and NPA were calculated by comparing the results of the conventional culture (reference method) with those of the BCID2. The conventional and BCID2 methods identified 86 pathogens and 95 pathogens from 40 specimens, respectively. The BCID2 method identified the same bacteria as the conventional method (+/+) as well as other bacterial species (+/−). See the text for details of the data analysis.

**Table 4:** Specimens identified as polymicrobial by the conventional method but identified differently by the BCID2 method (n = 9).

No.	ID by the conventional method	ID by the BCID2 method	
		Concordant microorganism	Discordant microorganism
1	*Staphylococcus lugdunensis* *Staphylococcus caprae	*Staphylococcus lugdunensis* *Staphylococcus* spp.	*S. epidermidis*
2	*Enterococcus faecium* *Klebsiella pneumoniae* *Citrobacter freundii* complex	*Enterococcus faecium* *Klebsiella pneumoniae* Enterobacterales	*Streptococcus* spp. *Klebsiella oxytoca* *Escherichia coli*
3	*Enterococcus faecalis* Klebsiella oxytoca **Aeromonas caviae	*Enterococcus faecalis* *Klebsiella oxytoca*	*Klebsiella pneumoniae*
4	*Escherichia coli* *Klebsiella oxytoca*	*Escherichia coli* *Klebsiella oxytoca*	*Pseudomonas aeruginosa* *Bacteroides fragilis*
5	*Escherichia coli* *Enterococcus faecium*	*Escherichia coli* *Enterococcus faecium*	*Bacteroides fragilis*
6	*Staphylococcus haemolyticus *Enterococcus faecalis*	*Staphylococcus* spp.	*Staphylococcus epidermidis* (*mec*A/C)
7	***Streptococcus agalactiae *Serratia marcescens*		*Streptococcus* spp.
8	*Enterococcus faecalis* *Escherichia coli* (NDM)	*Enterococcus faecalis* *Escherichia coli* (NDM)	*Pseudomonas aeruginosa*
9	*Enterobacter cloacae* complex *Klebsiella oxytoca*	*Enterobacter cloacae* complex *Klebsiella oxytoca*	*Stenotrophomonas maltophilia*

ID, species identification; NDM, New Delhi metallo-β-lactamase.

* This microorganism is not a target of the panel. *Staphylococcus caprae* and *Staphylococcus haemolyticus* both belong to the genus *Staphylococcus*. Therefore, this combination was determined to be concordant, based on the definitions of concordance and discordance (see main text).

** This microorganism is not a target of the panel, and the BCID2 method could not identify the isolate even at the genus level. Therefore, this combination was determined to be discordant, based on the definitions of concordance and discordance (see main text).

*** This microorganism is a target of the panel, and the BCID2 method was able to identify this isolate at the species level. Therefore, this combination was determined to be discordant, based on the definitions of concordance and discordance (see main text).

In the polymicrobial identification, we identified 98 microorganisms from 40 specimens. PPA ranged from 94% to 100% except for *S. agalactiae* (75%) and *Serratia marcescens* (50%), while NPA ranged from 96% to 100%, indicating that the BCID2 method demonstrated high performance in polymicrobial identification, whereas performance was lower compared with monomicrobial identification.

### Drug resistance

Among the isolates identified by the conventional method, 61 were found to be drug-resistant in antimicrobial susceptibility testing: 24 MRSA, 26 MRCNS, 10 ESBL-producing bacteria, and 1 carbapenem-resistant Enterobacteriaceae (CRE). The BCID2 method detected resistance in 57 isolates: all 24 MRSA were identified by the combined detection of *mec*A/C and MREJ, 22 of the 26 MRCNS were identified by *mec*A/C, all 10 ESBL producers were identified by CTX-M, and the single CRE was identified by NDM (Table 5). Thus, full concordance between the conventional and BCID2 methods was observed for MRSA (*mec*A/C and MREJ), ESBL producers (CTX-M), and CRE (NDM). Discordance was found in 4 CNS isolates. Accordingly, the total concordance rate was 93.4% (57 of 61), according to our concordance criteria.

**Table 5:** Detection of resistance genes by the BCID2 method in resistant microorganisms confirmed using the conventional method.

Resistance gene	No. of isolates	No. of isolates with agreement	Concordance rate (%)
*mec*A/C and MREJ	24	24	100
*mec*A/C	26	22	84.6
CTX-M	10	10	100
NDM	1	1	100
total	61	57	93.4

MREJ, SCC*mec* right-extremity junction; NDM, New Delhi metallo-β-lactamase.

## Discussion

In the present study, we compared species identification results between the BCID2 method and the conventional method, using specimens from blood culture-positive bottles. The overall concordance rate was 91.0% (183 of 201). Of the 201 specimens, 161 were identified as single microorganisms, using the conventional method. When using the BCID2 method, 152 specimens were also identified as monomicrobial, and 9 were identified as polymicrobial. The concordance rate was 100% when the monomicrobial pathogen was identified by both methods, but the overall concordance rate for monomicrobial identification was 94.4% (152 of 161). Similar results were obtained in previous studies [9–16]. Thus, the BCID2 method exhibited an excellent ability to identify microorganisms when the results indicated the presence of a single species. We identified 40 polymicrobial specimens, using the conventional method. Of the 40 specimens, 31 (77.5%) showed concordance between the conventional and BCID2 methods. The lower concordance rate for polymicrobial specimens than for monomicrobial specimens is in line with previous reports [9,11–13,17].

When both PPA and NPA were calculated, they were high enough to demonstrate the high performance of the BCID2 method. These results are also in line with previous reports [13,18].

In the present study, 18 specimens (9.0%) were identified with discordant identification (Tables 2 and 4). A higher discordant rate has been reported previously [9,11,13,15]. In all 18 specimens, the BCID2 method identified additional microorganism(s) beyond those identified by the conventional method. In three specimens, the BCID2 method was unable to identify the microorganisms detected by the conventional method (Table 4 #3, #6, and #7). *A. caviae*, identified by the conventional method, is an off-panel microorganism (Table 4 #3), and the BCID2 method could not identify it even at the genus level. Why the BCID2 method failed to identify *Streptococcus agalactiae*, *Enterococcus faecalis*, and *Serratia marcescens* (Table 4 #6 and #7) is unknown, but it may be due to limitations of the BCID2 method when analyzing polymicrobial infections.

One of the additional microorganisms identified by the BCID2 method was *S. epidermidis* (6 cases). Because species in CNS form similar colonies, it is possible that it was misidentified in previous reports [9,13,17]. Like CNS, *K. oxytoca* and *K. pneumoniae* are also species that form similar colonies. This may have complicated the accurate picking of their colonies (Table 2 #9 and Table 4 #2 and #3). *C. tropicalis* and *C. parapsilosis* are closely related species, and mixed infections that are difficult to distinguish can only be considered a single species (Table 2 #6 and #7) [19]. In addition, cross-reactivity between *C. tropicalis* and *C. parapsilosis* and the presence of non-viable *C. tropicalis* nucleic acid/DNA fragments in specific lots of blood-culture bottles—we used two relevant lots in the present study—have been reported by bioMérieux (Table 2 #6, #7, and #8) (https://www.accessdata.fda.gov/scripts/ires/index.cfm?Product=204997). Moreover, the presence of nucleic acid from non-viable *E. coli* has been identified in specific lots of blood-culture bottles [10].

*Bacteroides fragilis* was detected by the BCID2 method (Table 4 #4 and #5). Because these specimens were polymicrobial, it was difficult to detect anaerobic microorganisms by microscopic examination [9]. Furthermore, anaerobic culture is not performed if the conventional method does not indicate the presence of anaerobic microorganisms. *Pseudomonas aeruginosa* (Table 4 #4 and #8) and *Stenotrophomonas maltophilia* (Table 4 #9) were also identified by the BCID2 method. Although *Pseudomonas aeruginosa* and *Stenotrophomonas maltophilia* were identified alongside *Enterobacterales*, *Enterobacterales* proliferated more rapidly in culture; therefore, the presence of these pathogens might have been overlooked when using the conventional method. The other discordantly identified microorganisms in polymicrobial specimens are unclear, possibly due to constraints in the analysis of polymicrobial infections. Although the BCID2 method is capable of highly sensitive detection, which is one of its major advantages, it is necessary to determine whether the identified bacteria are actually pathogenic if they are not detected using the conventional method. In other words, it must be determined whether each bacterium is a pathogen when the BCID2 method shows polymicrobial specimens.

The following case may provide some insight into the performance of the BCID2 method. In the specimen in which *E. faecalis* was identified by the conventional method and *E. faecalis* and *S. lugdunensis* were identified using the BCID2 method (Table 2 #5), the patient was treated with ampicillin, based on the result of the conventional method but did not improve. A blood culture was taken again 3 days later. *S. lugdunensis* (MRCNS) was identified using the conventional method, and daptomycin was administered instead of ampicillin. The patient’s condition subsequently improved. It is possible that *S. lugdunensis* could not be identified using the conventional method in the first blood culture because the amount of *S. lugdunensis* in the culture bottle was low or its growth was masked.

When comparing drug-resistance detection between the BCID2 and conventional methods, overall concordance was excellent (93.4% [57 of 61]) (Table 5). The concordance rates were 100% for MRSA (*mec*A/C and MREJ), ESBL producers (CTX-M), and CRE (NDM) but 84.6% for MRCNS (*mec*A/C). Similar concordance rates have been reported previously [6,20]. In the present study, 26 MRCNS were identified using the conventional method. Of these, 2 were identified as *S. caprae* (MRCNS), 1 as *S. hominis* (MRCNS), and 1 as *S. capitis* (MRCNS). Because the BCID2 method does not report *mec*A/C for CNS except for *S. epidermidis* and *S. lugdunensis* [10], the resistance genes in these 4 MRCNS were not evaluated.

This study has several limitations. First, the BCID2 method can detect 33 bacterial species, corresponding to approximately 80% of the species identified in positive blood cultures in the last 10 years at our hospital. BCID2 can even identify bacterial species that do not grow in culture. This is probably due to its high sensitivity, but clinicians must consider whether each bacterium is a pathogen, particularly in the case of polymicrobial identification. Second, BCID2 can detect 10 different resistance genes. However, in the present study, only four resistance genes—MRCNS (*mec*A/C), MRSA (*mec*A/C and MREJ), ESBL (CTX-M), and CRE (NDM)—were detected. We cannot draw any conclusions about performance for the other 6 resistance genes. Third, the BCID2 method does not report *mec*A/C for CNS except for *S. epidermidis* and *S. lugdunensis* [12], although CNS identification at the species level would not have a clinical impact. Fourth, the BCID2 method cannot identify *Enterococcus* spp. other than *E. faecalis* and *E. faecium*. It has been reported that non-*E. faecalis* and *E. faecium Enterococcus* spp. account for 6% and 21% of enterococcal infections, respectively [21,22]. Careful observation is required when enterococcal infection is suspected. Finally, there are other resistance genes that are not included in the BCID2 panel, such as SHV and TEM in ESBL-producing organisms [13].

## Conclusions

This study demonstrated that the BCID2 method is a reliable method for species identification and resistance-gene detection in bloodstream infections. Species identification was sufficiently accurate to be acceptable even for polymicrobial identification, but some improvements are necessary to increase the number of identifiable bacteria and to enable accurate identification at the species level. In addition, we must determine whether each bacterium is a pathogen when the BCID2 method identifies a polymicrobial specimen. Regarding the detection of resistance genes, a highly precise detection capability was shown. To detect more-resistant bacteria, it is important to include more resistance genes, such as AmpC, in the panel. Additional validation and further improvements would accelerate species identification and influence the selection of appropriate antimicrobial agents.
